# A case report of primary biliary cholangitis combined with ankylosing spondylitis

**DOI:** 10.1097/MD.0000000000035655

**Published:** 2023-10-13

**Authors:** Chunfeng Hou, Chunfeng Ren, Luan Luan, Shujie Li

**Affiliations:** a Department of Rheumatology, Jining No.1 People’s Hospital, Jining, China.

**Keywords:** 99Tc-MDP, ankylosing spondylitis, case report, primary biliary cholangitis, ursodeoxycholic acid

## Abstract

**Rationale:**

A chronic autoimmune liver disease known as primary biliary cholangitis (PBC) that selectively destructs small intrahepatic biliary epithelial cells and may result in biliary cirrhosis and eventually liver transplantation or death. PBC is associated with various other extrahepatic autoimmune diseases; however, the combination of PBC with ankylosing spondylitis has been rarely reported in the literature. Here, we reported a case of PBC with ankylosing spondylitis to improve our understanding of such coexistence and provide new ideas for the treatment of such patients.

**Patient concerns:**

A 54-year-old man was presented to the Department of Rheumatology because of an abnormal liver function test for 7 years, chest and back pain for 1 year, and low back pain for 2 months.

**Diagnoses:**

Primary biliary cholangitis, ankylosing spondylitis, and old pulmonary tuberculosis.

**Interventions:**

The patient refused to use nonsteroidal anti-inflammatory drugs, conventional synthetic disease-modifying antirheumatic drugs, and biologic disease-modifying antirheumatic drugs; thus, he was treated with methylenediphosphonate (99Tc-MDP) and ursodeoxycholic acid (UDCA).

**Outcomes:**

The patient achieved remission with UDCA and 99Tc-MDP therapy.

**Lessons:**

In the treatment of PBC combined with other disorders, the characteristics of different diseases should be considered. The patient reported herein was treated with 99Tc-MDP and UDCA, and his condition improved; thus, we consider 99Tc-MDP to be an effective treatment. Furthermore, in line with the current understanding of the pathogenesis of PBC and ankylosing spondylitis, we hypothesize that interleukin-17 inhibitor is an effective treatment for such patients.

## 1. Introduction

Primary biliary cholangitis, nonsuppurative destructive cholangitis, is the archetype of an autoimmune disease, whose pathogenesis remains largely obscure.^[1]^ Primary biliary cholangitis (PBC) selectively destructs small intrahepatic biliary epithelial cells and may result in biliary cirrhosis, ultimately leading to liver transplantation or death.^[2,3]^ It is the most prevalent autoimmune disease in females, with most cases occurring in those aged 40 to 60 years.^[4,5]^ PBC patients commonly experience fatigue, pruritus, hyperlipidemia, and osteopenia, with fatigue or pruritus affecting more than 50% of these patients.^[6]^ The serological tests mainly show elevated alkaline phosphatase (ALP) and positive anti-mitochondrial antibody (AMA) levels or specific antinuclear antibodies(anti-gp210, anti-sp100, and anti-PML).^[7,8]^ Histologically, in PBC patients, progressive small bile duct injury that is facilitated by a coordinated and serial development of effector pathways at distinct disease phases is observed.^[9–11]^ A recent comprehensive investigation found that 28.3% of PBC patients had extrahepatic autoimmune diseases, of which only 0.2% had concurrent ankylosing spondylitis (AS).^[12]^ The disturbed balance of the innate immune system and acquired immune system in response to environmental factors results in AS, a chronic inflammatory rheumatic disease.^[13]^ The spine and sacroiliac joints are affected by AS, which causes inflammatory back pain, thus decreasing the quality of life of patients and increasing the burden on patients and society.^[14]^ The association between PBC and AS has rarely been established, and only 3 cases have been reported to date.^[15–17]^ As far as we are aware, no reports of PBC with AS in China exist. Our current case report presents an uncommon case of a patient who had PBC and developed AS. Different from the previously reported cases, the patient suffered from PBC first and then developed AS; he was administered ursodeoxycholic acid (UDCA) and methylenediphosphonate (99Tc-MDP), and his condition improved.

## 2. Case report

**Figure 1.**
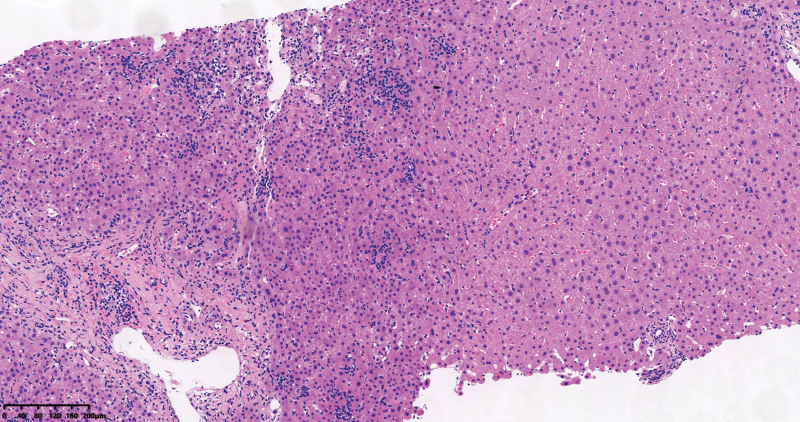
The hematoxylin–eosin staining (×100) of the liver tissue revealed that a part of the portal area was enlarged with fibrous tissue hyperplasia, and there was portal lymphocytic infiltration and bile duct damage.

A 54-year-old man had elevated ALT, AST, ALP, and gamma-glutamyl transferase levels for 7 years. He took oral hepatoprotective medications intermittently, and his condition was poorly controlled. Despite visiting several hospitals, his diagnosis remained unclear. Therefore, a liver biopsy was performed in April 2019. The hematoxylin–eosin staining of the liver tissue revealed that a part of the portal area was enlarged with fibrous tissue hyperplasia, and there was portal lymphocytic infiltration and bile duct damage; these findings were compatible with those of PBC (Fig. 1). Therefore, UDCA was used to treat the patient after a PBC diagnosis. Since then, he had taken UDCA 500 mg twice a day, and his liver function had been normal for the last 6 months. The patient had recurrent chest and back pain with morning stiffness more than half an hour 1 year ago, which could not be relieved by rest. Two months ago, he developed low back pain, with worsening chest and back pain, which was obvious while changing positions, coughing, and deep breathing; he occasionally woke up with pain at night. The patient had old pulmonary tuberculosis.

His physical examination showed that skin, mucous membranes, and sclera were not yellow; there was no tenderness on the sternum, no percussion pain in the liver area, and no joint swelling and tenderness; there was tenderness of the thoracic and lumbar spine spinous process, an occiput-to-wall distance of 1 cm, chest expansion (3cm), and a finger-to-floor distance of 4 cm; and the Patrick Faber test was negative for both sides.

**Figure 2.**
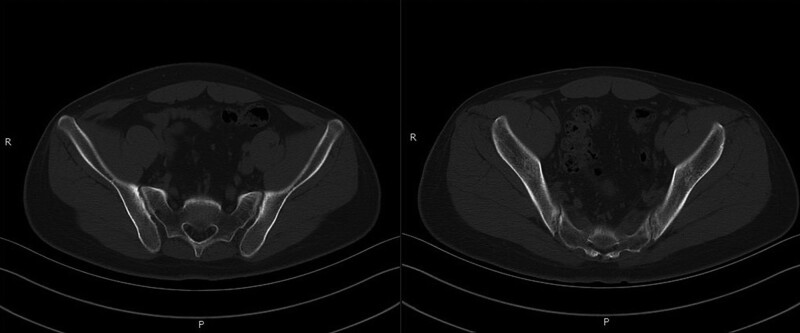
The surface of bilateral sacroiliac joints was rough and serrated, and the sacrum and the auricular surface of the ilium were thickened and uneven.

The auxiliary examination findings after admission were as follows: The serological tests for hepatitis virus, anti-smooth muscle antibody, antinuclear antibodies, anti-liver kidney microsomal antibody, anti-GP210, anti-sp100, and AMA were negative. The C-reactive protein level was elevated to 12.5 mg/L. The test for HLA-B27 was negative. Liver function, erythrocyte sedimentation rate, complete blood cell count, immunoglobulins, and complement and Rheumatoid factor were normal. The thoracic computed tomography showed an old lesion in the upper lobe of the left lung. The liver magnetic resonance imaging showed cirrhosis and splenomegaly. Sacroiliac joint computed tomography demonstrated bilateral sacroiliac arthritis (grade II–III) (Fig. 2).

The final diagnosis was primary biliary cholangitis, AS, and old pulmonary tuberculosis. We recommended interleukin (IL)-17 inhibition and prophylactic antituberculosis treatments. However, the patient refused to use nonsteroidal anti-inflammatory drugs, conventional synthetic disease-modifying antirheumatic drugs, and biologic disease-modifying antirheumatic drugs due to concerns about adverse drug effects; therefore, he was given only 99Tc-MDP 16.5 mg intravenous QD for 14 days and continuous administration of UDCA 500 mg twice a day. Fortunately, his chest, back, and low back pain and morning stiffness were reduced after treatment. After discharge, the patient continued taking UDCA, without pain in the chest, back, and low back. Two weeks later, he was again treated with 99Tc-MDP, and the liver function and inflammatory indicators were normal. The patient received 3 courses of 99Tc-MDP treatment at weeks 0 to 2, 6 to 8, and 12 to 14, respectively. Thereafter, the patient received 99Tc-MDP approximately every 6 months.

## 3. Discussion

PBC is an immune-mediated liver disease described by positive AMA and the damage of small-to-medium-sized intrahepatic bile ducts. It is a chronic disease that can advance to end-stage liver disease and its related complications.^[1]^ The disease was first described in a case report from 1851 that involved a 42-year-old woman with xanthomata and jaundice,^[18]^ it was called primary biliary cirrhosis in 1949.^[19]^ Primary biliary cirrhosis, however, was renamed primary biliary cholangitis in 2015 due to the fact that cirrhosis is present only when the disease has progressed.^[20,21]^ The etiology of PBC is unclear. Although a number of significant findings have greatly advanced our understanding of PBC, its pathogenesis is still mostly unknown. Numerous lines of evidence imply that genetic predisposition and environmental factors may serve as a background from which PBC emerges. The findings of numerous genome-wide association studies and the perceived higher incidence of PBC in siblings, particularly in monozygotic twins, have been linked to the function of genetic risk factors.^[22–25]^ Environmental stimuli including bacterial infection and exposure to xenobiotics are likely to cause loss of self-tolerance.^[9,26,27]^ Moreover, the abnormal immune response is known to be the vital driving force behind the progression of PBC.^[28–33]^ Numerous immune cell subsets, cytokines, and other mediators are associated with the onset, progression, and perpetuation of disease and vary according to the stage of the disease. Th17 cells, a subset of helper T cells, can produce high IL-17 levels.^[34]^ According to studies conducted on PBC patients, the Th17 signaling pathway has been implicated in the pathogenesis of PBC. Furthermore,Th17 skewing is prominently observed in patients with PBC at advanced stages with the intensive secretion of IL-23p19 by inflamed hepatocytes around IL-12Rβ2, IL-23R, and IFN-γ expressing degenerated cholangiocytes.^[31,35]^ The Th1/Th17 balance shifts toward the direction of Th17 in PBC patients at advanced stages; thus, we hypothesize that Th1 has been implicated in the occurrence of the disease, whereas Th17 is necessary for the perpetuation of ongoing pathology.^[31]^ In addition to immune cells, a plethora of evidence suggests that biliary epithelial cells are active participants in the onset and perpetuation of autoimmunity in PBC rather than being innocent victims.^[1]^ A growing number of studies discovered that the peripheral blood IL-17 levels in PBC patients were substantially increased in contrast with those in normal subjects.^[31,36–38]^ Moreover, the expression of IL-17 mRNA was significantly higher in the peripheral blood mononuclear cells of the PBC group in contrast with that of the healthy control group^[39]^; it is speculated that IL-17 inhibition is effective in PBC treatment. According to the 2018 EASL Clinical Practice Guidelines, the PBC can be diagnosed if there is no extrahepatic biliary blockage and no liver-related comorbidities, and at least 2 of the following requirements are fulfilled; Higher ALP and gamma-glutamyl transferase levels; Presence of AMA or other PBC specific autoantibodies (sp100 or gp210) if AMA is negative; and Histologic evidence of PBC (nonsuppurative destructive cholangitis and damage of the interlobular bile ducts).^[40]^ The only 2 drugs that the US Food and Drug Administration has currently approved for the treatment of PBC are UDCA and obeticholic acid.^[41]^ Other targeted drugs, including PPAR agonists and those that target FGF19, are being developed in addition to UDCA and obeticholic acid. With the discovery of further mechanisms, more targets are incorporated in the selected ranks, including CX3CL1 and NOX1/NOX4.^[42]^

PBC patients immunological abnormalities generally result in a number of concomitant autoimmune diseases. Sjogren syndrome is the comorbidity that PBC patients display most frequently among all comorbidities.^[43,44]^ PBC patients have also been shown to have autoimmune thyroid disease, systemic lupus erythematosus, scleroderma, and celiac disease.^[45–49]^ However, PBC combined with AS has rarely been reported. The axial skeleton is affected by the prevalent inflammatory rheumatic disease known as AS, which results in characteristic inflammatory back pain, thus leading to impairments of structure and function and a deterioration in the quality of life.^[13]^ The underlying mechanism of AS is believed to be autoimmune or autoinflammatory.^[50]^ An increasing number of studies have recently explored the pathogenesis and etiology, imaging techniques, and treatment in AS.^[51–55]^ AS is a disease that is mediated by numerous cell types and pathogenic pathways. The pathogenesis of AS can be attributed to the imbalance between Th17 and Tregs and Th1 and Th2, which further affirms that AS is caused by the imbalance between the innate immune system and the acquired immune system.^[56]^ Moreover, a significant contributor to the pathogenesis of spondyloarthritis is cytokine signaling via the IL-17A pathway.^[57,58]^ Patients with AS have been found to have elevated circulating IL-17 levels in contrast with healthy controls.^[59]^ Furthermore, the number of pathogenic TH17, TH22 cells, and IL-17-secreting γδ cells in the peripheral blood of AS patients also increase and accumulate in the joints.^[60–62]^ Upon stimulation, Th17 cells secrete IL-17, which is primarily a pro-inflammatory factor that causes tissue inflammation by generating a number of pro-inflammatory cytokines and chemokines, along with profibrotic effects, contributing to organ fibrosis.^[63]^ The monoclonal antibody that targets IL-17A is effective in treating AS symptoms and may be effective in treating PBC. Y. Xu et al^[64]^ confirmed the remarkable efficacy of 99Tc-MDP in a large number of patients with refractory AS and that 99Tc-MDP was safe in clinical application; therefore, the patient was administered 99Tc-MDP treatment.

## 4. Conclusion

In summary, according to the present understanding of the pathogenesis of PBC and AS, it is speculated that Th17 plays a role in the combination of these 2 diseases, which requires further research and validation. However, there are relatively few cases of such comorbidities and making it difficult to conduct large-scale case-control studies. There are few reports of PBC combined with AS to date and the accumulated data and information are limited; however, it is believed that the incidence of such patients may not be accidental. Therefore, more attention should be paid to these 2 diseases in clinical practice, and the association between these 2 diseases need to be further studied to form an early diagnosis, initiate early treatment, and have better long-term outcomes.

## Author contributions

**Conceptualization:** Chunfeng Hou, Shujie Li.

**Data curation:** Chunfeng Ren.

**Investigation:** Chunfeng Hou.

**Writing – original draft:** Chunfeng Hou.

**Writing – review & editing:** Luan Luan, Shujie Li.
